# Fitness-Tracker Assisted Frailty-Assessment Before Transcatheter Aortic Valve Implantation: Proof-of-Concept Study

**DOI:** 10.2196/19227

**Published:** 2020-10-15

**Authors:** Markus Mach, Victoria Watzal, Waseem Hasan, Martin Andreas, Bernhard Winkler, Gabriel Weiss, Andreas Strouhal, Christopher Adlbrecht, Georg Delle Karth, Martin Grabenwöger

**Affiliations:** 1 Division of Cardiac Surgery Department of Surgery Medical University of Vienna Vienna Austria; 2 Department of Cardio-Vascular Surgery Hospital Hietzing and Karl Landsteiner Institute for Cardio-Vascular Research Vienna Austria; 3 Faculty of Medicine Imperial College London London United Kingdom; 4 Department of Cardiology Vienna North Hospital - Clinic Floridsdorf and Karl Landsteiner Institute for Cardiovascular and Critical Care Research Vienna Austria; 5 Medical Faculty Sigmund Freud University Vienna Austria

**Keywords:** frailty, activity, fitness, tracker, transcatheter aortic valve implantation, transcatheter aortic valve repair

## Abstract

**Background:**

While transcatheter aortic valve replacement (TAVR) has revolutionized the treatment of aortic valve stenosis, wearable health-monitoring devices are gradually transforming digital patient care.

**Objective:**

The aim of this study was to develop a simple, efficient, and economical method for preprocedural frailty assessment based on parameters measured by a wearable health-monitoring device.

**Methods:**

In this prospective study, we analyzed data of 50 consecutive patients with mean (SD) age of 77.5 (5.1) years and a median (IQR) European system for cardiac operative risk evaluation (EuroSCORE) II of 3.3 (4.1) undergoing either transfemoral or transapical TAVR between 2017 and 2018. Every patient was fitted with a wrist-worn health-monitoring device (Garmin Vivosmart 3) for 1 week prior to the procedure. Twenty different parameters were measured, and threshold levels for the 3 most predictive categories (ie, step count, heart rate, and preprocedural stress) were calculated. Patients were assigned 1 point per category for exceeding the cut-off value and were then classified into 4 stages (no, borderline, moderate, and severe frailty). Furthermore, the FItness-tracker assisted Frailty-Assessment Score (FIFA score) was compared with the scores of the preprocedural gait speed category derived from the 6-minute walk test (GSC-6MWT) and the Edmonton Frail Scale classification (EFS-C). The primary study endpoint was hospital mortality.

**Results:**

The overall preprocedural stress level (*P=.*02), minutes of high stress per day (*P=.*02), minutes of rest per day (*P=.*045), and daily heart rate maximum (*P=.*048) as single parameters were the strongest predictors of hospital mortality. When comparing the different frailty scores, the FIFA score demonstrated the greatest predictive power for hospital mortality (FIFA area under the curve [AUC] 0.844, CI 0.656-1.000; *P=.*048; GSC-6MWT AUC 0.671, CI 0.487-0.855; *P=.*42; EFS-C AUC 0.636, CI 0.254-1.000; *P=.*44).

**Conclusions:**

This proof-of-concept study demonstrates the strong predictive performance of the FIFA score compared to that of the conventional frailty assessments.

## Introduction

Since the first transcatheter aortic valve replacement (TAVR) performed in 2002 by Alain Cribier, little development has taken place in the field of frailty assessment [1]. Despite being referred to as “the most important patient characteristic not included in current risk models” in the Valve Academic Research Consortium-2 (VARC-2) consensus document, the frailty assessment tool that is the most appropriate for clinical practice remains debated [2,3]. While existing objective performance measures such as gait speed or grip strength lack the specificity to distinguish between frail and nonfrail patients when used on their own, the development of multimodal and TAVR-specific frailty scores has yielded promising approaches that outperform the most widely used risk scores [4-9]. However, as most of these conventional frailty assessment tools are relatively time-consuming and resource-consuming, frailty is still often assessed subjectively based upon either an “eyeball test,” which is an “end-of-the-bed” assessment, or not measured at all in clinical practice [5].

Wearable health-monitoring devices have already been used for patient-related basic measurements in different specialties. Several studies have proven that such devices can reliably predict flares in patients with rheumatic diseases or reverse remodeling in patients with heart failure treated with resynchronization therapy [2-4]. These mediums, therefore, represent a potential platform where measurements relevant to the patients’ health status and subsequent intervention can be collected without significantly interfering with the activities of both patients and clinical staff. The aim of this study was to develop a preprocedural frailty assessment based on data measured by a wearable health-monitoring device.

## Methods

### Patient Population and Study Design

The present open-label, nonrandomized proof-of-concept study features permanent preprocedural activity monitoring with a wearable health-monitoring device (Vivosmart3, software version 2.9-5.10, Sensor Hub software version 6.3., Garmin) during the week prior to the intervention. All patients treated between March and December 2018 at the Heart Center Hietzing (Vienna, Austria) were invited to participate and they provided written informed consent. Baseline patient characteristics, as well as procedural and outcome data, were obtained from the VIennaCardioThOracic Aortic Valve RegistrY. The Ethics Committee of the City of Vienna approved the study (protocol EK-048-0318).

The indication for TAVR was assessed by a multidisciplinary Heart Team, consisting of cardiologists, cardiac surgeons, anesthetists, radiologists, and geriatricians. Intermediate-risk or high-risk patients with severe aortic stenosis were subject to evaluation and were included, conforming to the current guidelines of the European Association of Cardio-Thoracic Surgery [5]. Hence, inclusion and exclusion criteria for the trial were the same as those for the procedure itself. No patient had physical limitations preventing him/her from wearing an activity tracker. Surgical risk stratification was based on the following risk algorithms: the logistic European system for cardiac operative risk evaluation (EuroSCORE), EuroSCORE II, and the Society of Thoracic Surgeons (STS) score [6-8]. To determine the prevalence of frailty, the Edmonton Frail Scale (EFS) was used and the 6-minute walk test (6MWT) was performed prior to the intervention [9,10]. The primary endpoint of the study was in-hospital all-cause mortality. The evaluation and documentation of the postprocedural outcome data followed the VARC-2 criteria [11].

### Frailty Assessment

The EFS includes an objective evaluation of the patient’s health status as well as the quality of life and clinical frailty. It comprises 5 categories: 0-5 points, not frail; 6-7 points, vulnerable; 8-9 points, mild frailty; 10-11 points, moderate frailty; and >12 points, severe frailty [9]. As an additional frailty assessment tool, the 6MWT was performed in the same visit in the outpatient department. We distinguished between very slow walkers (<0.5 m/s), slow walkers (≥0.5 m/s), normal walkers (>0.83 m/s), and patients, in whom the 6MWT was not feasible because of general weakness, being bedridden, being wheelchair-bound, or pain [12].

### Wearable Health-Monitoring Device

The Vivosmart 3 continuously measures daily physical activity. It assesses the daily step count, distance covered (in kilometers), calories burned, the time spent in different stress levels, the hours and depth of sleep, the minimum and maximum heart rate, and the number of flights of stairs climbed. Several reports have validated the accuracy of the heart rate measurement by Garmin Vivosmart devices with simultaneous electrocardiogram readings [13,14], and the device has shown excellent test-retest reliability as well as optimal step count accuracy at low and moderate walking speeds [15]. The stress level measurement calculated by the device is based on the analysis of the heart rate variability and it is a good reflector of autonomic activity. Real-life heart rate measurements using photoplethysmography are combined with mathematical modeling and algorithms to infer the present stress level of the patient wearing the monitoring device [16].

The waterproof device was worn for at least one week prior to the procedure around the wrist of the nondominant hand to avoid additional step count through repetitive gestures during daily activities. Patients were encouraged to follow their regular routines and activities while continuously wearing the device and they were instructed not to remove it temporarily for bathing, swimming, or sleeping.

On the day of admission, the device was removed, and the activity data were uploaded via password-encrypted Bluetooth transfer to anonymous accounts in the Garmin Connect app (version 3.22.0.1-4.20). The device had no GPS-tracking function, thereby ensuring maximum data security.

### Fitness-Tracker Assisted Frailty-Assessment

The objective of this study was to develop a modern, easy-to-use, time-saving, and resource-saving frailty assessment method that allows accurate prediction of adverse outcomes after TAVR. The daily output was used to calculate the weekly average values, excluding the incomplete activity data available from the first and last day of monitoring. Threshold levels in 3 predefined categories (ie, heart rate, preprocedural stress, and walking) were calculated. The patients were assigned 1 point per category when exceeding (in categories with positive correlation) or subceeding (in categories with negative correlation) the threshold levels and then grouped into 4 categories (0, no frailty; 1, mild frailty; 2, moderate frailty; 3, severe frailty). Sleep pattern parameters were assessed for their discriminatory power but not included in the FItness-tracker assisted Frailty-Assessment (FIFA) score, as currently, no evidence exists that such parameters are correlated with adverse postoperative outcomes.

### Statistical Analysis

Continuous data were expressed as mean (SD) or median (IQR) depending on the normal distribution. The comparison of continuous data between groups was performed using the Kruskal-Wallis test (H test) or univariate analysis when appropriate. A chi-square or Fisher exact test was performed to compare categorical data. Posthoc testing was performed using the Bonferroni corrected z test, Duncan, or Scheffé test depending on variance homogeneity and sample size.

For the development of the FIFA score, different parameters identifying frail patients and positively predicting hospital mortality were determined by receiver operating characteristic (ROC) analysis. The discriminatory ability was assessed via the area under the curve (AUC). Threshold values were calculated retrospectively with the Youden Index *(J = sensitivity + specificity –1)*. The different frailty and risk assessment tools were then again compared for their predictive power of hospital mortality with ROC and AUC analysis. The alpha level was set at less than *.*05. All tests were two-tailed. Statistical calculations were performed using the SPSS statistical software version 24.0 (IBM Corp).

## Results

### Baseline Patient Characteristics

The baseline characteristics of the cohort are outlined in Table 1. In total, 50 patients (22 women, 44%) with a mean (SD) age of 77.5 (5.1) years were included. The surgical risk profile of the cohort was as follows: mean (SD) EuroSCORE II, 3.3 (4.1) and median (IQR) STS score, 2.9 (2.3). The patients were stratified according to their FIFA score classification (no, mild, moderate, or severe frailty). There was a strong correlation between the FIFA score and the baseline serum albumin level, as more frail patients had significantly lower albumin levels (*P=.*005). Apart from the serum albumin levels, the baseline characteristics, including echocardiographic parameters, were similar among the groups.

**Table 1:** Baseline characteristics of the cohort.

Characteristics			Overall, N=50		No frailty, n=11		Mild frailty, n=10		Moderate frailty, n=21		Severe frailty, n=8		*P* value^a^
**Demographics**													
	Age (years), mean (SD)	77.5 (5.1)		78.0 (3.3)		76.4 (4.8)		78.7 (4.9)		74.9 (7.2)		.28	
	Female, n (%)	22 (44)		5 (46)		6 (60)		8 (38)		3 (38)		.69	
	Body mass index (kg/m^2^), mean (SD)	27.2 (5.3)		28.5 (3.9)		27.3 (7.5)		26.1 (4.3)		28.0 (6.7)		.63	
	Serum albumin (g/dl), mean (SD)	40.3 (3.3)		42.7 (2.0)		38.6 (3.9)		40.4 (2.2)		38.1 (3.9)		*.005*	
**Risk scores**													
	EuroSCORE II^b^, median (IQR)	3.3 (4.1)		4.9 (6.4)		2.6 (1.7)		3.3 (5.2)		3.4 (2.6)		.32	
	Logistic EuroSCORE, mean (SD)	12.0 (8.1)		16.3 (11.0)		11.3 (6.4)		11.6 (7.4)		8.2 (5.4)		.17	
	STS^c^ score, median (IQR)	2.9 (2.3)		2.7 (1.7)		2.4 (2.9)		3.6 (3.6)		2.8 (1.6)		.34	
**Comorbidities**													
	Hypertension, n (%)	47 (94)		11 (100)		9 (90)		19 (91)		8 (100)		.58	
	Diabetes mellitus (IDDM)^d^, n (%)	19 (38)		2 (18)		5 (50)		9 (43)		3 (38)		.59	
	Atrial fibrillation, n (%)	26 (52)		8 (73)		4 (40)		10 (48)		4 (40)		.45	
	Peripheral vascular disease, n (%)	23 (46)		5 (46)		3 (30)		11 (52)		4 (50)		.69	
	COPD^e^, n (%)	15 (30)		2 (18)		5 (50)		5 (24)		3 (38)		.09	
	Cerebrovascular accident, n (%)	10 (20)		2 (18)		1 (10)		6 (29)		1 (13)		.76	
	Creatinine (mg/dL), median (IQR)	1.1 (0.7)		1.1 (0.6)		0.9 (0.5)		1.2 (1.0)		1.5 (0.8)		.16	
	Prior myocardial infarction, n (%)	6 (12)		2 (18)		1 (10)		3 (14)		0 (0)		.65	
	Prior PCI^f^, n (%)	15 (30)		6 (55)		0 (0)		5 (24)		4 (50)		*.03*	
	Previous CABG^g^, n (%)	6 (12)		3 (27)		1 (10)		2 (10)		0 (0)		.30	
	Previous valve surgery, n (%)	4 (8)		2 (18)		0 (0)		2 (10)		0 (0)		.37	
	sPAP^h^, mean (SD)	47.1 (15.8)		48.6 (20.9)		36.3 (3.2)		47.0 (14.5)		51.8 (14.0)		.49	
	LVEF%^i^, mean (SD)	50.0 (15.5)		54.3 (13.1)		63.5 (12.1)		41.7 (16.0)		50.8 (12.2)		.10	

^a^Statistically significant *P* values are italicized in the table.

^b^EUROScore: European system for cardiac operative risk evaluation.

^c^STS: Society of Thoracic Surgeons.

^d^IDDM: insulin-dependent diabetes mellitus.

^e^COPD: chronic obstructive pulmonary disease.

^f^PCI: percutaneous coronary intervention.

^g^CABG: coronary artery bypass graft.

^h^sPAP: systolic pulmonary artery pressure.

^i^LVEF: left ventricular ejection fraction.

### Procedural Outcomes and Adverse Events

The procedural outcomes are shown in Table 2. The procedural time, total intensive care unit hours, and hospital stay did not differ between the frailty stages. The same applied to adverse events, as seen in Table 3.

**Table 2:** Procedural characteristics and outcomes in the different groups of the cohort.

Procedures			Overall, N=50			No frailty, n=11		Mild frailty, n=10			Moderate frailty, n=21		Severe frailty, n=8		*P* value
**Prosthesis used, n (%)**														.73	
	Sapien XT	4 (8)		2 (18)			0 (0)		2 (10)			0 (0)			
	Sapien 3	27 (54)		6 (55)			6 (60)		10 (48)			5 (63)			
	Symetis Acurate	15 (30)		3 (27)			3 (30)		6 (29)			3 (38)			
	Core Valve Evolut	4 (8)		0 (0)			1 (10)		3 (14)			0 (0)			
Total ICU^a^ hours, median (IQR)			22.0 (29.0)		32.0 (48.0)			23.0 (33.0)		21.0 (4.0)			18.5 (281.0)		.21
Length of stay in days, median (IQR)			13.0 (10.0)		15.0 (13.0)			11.0 (12.0)		13.0 (8.0)			17.0 (20.0)		.42

^a^ICU: intensive care unit.

**Table 3:** Adverse events in the different groups of the cohort.

Types of adverse events			Overall, N=50		No frailty, n=11		Mild frailty, n=10		Moderate frailty, n=21		Severe frailty, n=8		*P* value
**Cerebrovascular events, n (%)**												.70	
	Transient ischemic attack	2 (4)		0 (0)		0 (0)		1 (5)		1 (13)			
	Major stroke	1 (2)		0 (0)		0 (0)		1 (5)		0 (0)			
**Access site complication, n (%)**												.52	
	Minor access complication	3 (6)		1 (9)		1 (10)		1 (5)		0 (0)			
	Major access complication	1 (2)		0 (0)		1 (10)		0 (0)		0 (0)			
	Myocardial infarction	2 (4)		0 (0)		0 (0)		1 (5)		1 (13)		.49	
	Postoperative pacemaker	7 (14)		2 (18)		2 (20)		2 (10)		1 (13)		.84	
**Postoperative acute kidney injury, n (%)**												.43	
	Stage I	6 (12)		0 (0)		1 (10)		3 (14)		2 (25)			
	Stage II	0 (0)		0 (0)		0 (0)		0 (0)		0 (0)			
	Stage III	2 (4)		0 (0)		0 (0)		2 (10)		0 (0)			
**Postoperative bleeding, n (%)**												.43	
	Minor bleeding	7 (14)		3 (27)		1 (10)		2 (10)		1 (13)			
	Major bleeding	2 (4)		1 (9)		0 (0)		0 (0)		1 (13)			
	Hospital mortality	3 (6)		0 (0)		0 (0)		1 (5)		2 (25)		.09	

### Discriminatory Power of Health-Monitoring Device–Generated Measurements as Predictors of Hospital Mortality

The overall preprocedural stress level (AUC 0.915, CI 0.795-1.000; *P=.*02), the minutes spent in high stress (AUC 0.908, CI 0.801-1.000; *P=.*02), the minutes at rest (AUC 0.848, CI 0.711-0.984; *P=.*045), and the heart rate maximum (AUC 0.844, CI 0.717-0.97; *P=.*048) as individual parameters were the strongest predictors of hospital mortality in the ROC analysis. High preprocedural stress levels, long periods spent in high stress, and an elevated maximum heart rate correlated positively with hospital mortality, whereas the amount of time at rest had an inverse correlation with hospital mortality (Table 4).

**Table 4:** Receiver operating characteristic analysis and hospital mortality.

Parameters				Area under the curve		Confidence interval				*P* value^a^
						Lower		Upper		
**Tracker parameters**										
	**Heart rate**									
		Heart rate minimum^b^	0.688		0.435		0.941		.28	
		Heart rate maximum^b^	0.844		0.717		0.971		*.048*	
	**Preprocedural stress**									
		Stress level at rest^c^	0.848		0.711		0.984		*.045*	
		Stress level, high^b^	0.908		0.801		1.000		*.02*	
		Overall stress level^b^	0.915		0.795		1.000		*.02*	
	**Walking**									
		Step count^c^	0.518		0.069		0.966		.92	
		Walking distance^c^	0.525		0.076		0.974		.89	
	**Sleeping**									
		Sleep state, deep^c^	0.560		0.085		1.000		.95	
		Sleep, light^b^	0.457		0.059		0.856		.81	
		Sleep, awake^b^	0.532		0.113		0.951		.85	
		Sleep, in total^c^	0.468		0.071		0.865		.95	
**Risk scores**										
	EuroSCORE^d^ II^b^			0.624		0.479		0.769		.47
	Logistic EuroSCORE^b^			0.461		0.241		0.681		.82
	STS^e^ score^b^			0.376		0.198		0.554		.47
**Frailty scores**										
	Gait speed classification (6MWT^f^)^b^			0.671		0.487		0.855		.42
	EFS^g^ classification^b^			0.636		0.254		1.000		.44
	FIFA^h^ score			0.844		0.656		1.000		*.048*

^a^Statistically significant *P* values are italicized in the table.

^b^Positive correlation with hospital mortality.

^c^Negative correlation with hospital mortality.

^d^EUROScore: European system for cardiac operative risk evaluation.

^e^STS: Society of Thoracic Surgeons.

^f^6MWT: 6-minute walk test.

^g^EFS: Edmonton Frail Scale.

^h^FIFA: FItness-tracker assisted Frailty-Assessment.

### Comparison of the Incremental Predictive Value of Frailty Scores

**Figure 1:**
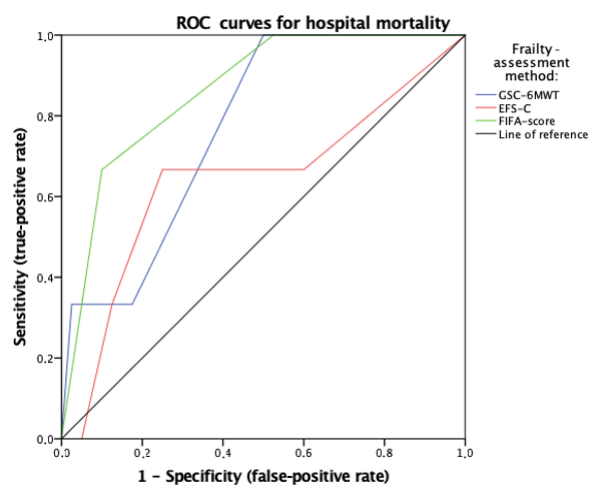
Receiver operating characteristic curves predicting hospital mortality. ROC: receiver operating characteristic; GSC-6MWT: gait speed category derived from the 6-minute walk test; EFS-C: Edmonton Frail Scale classification; FIFA: FItness-tracker assisted Frailty-Assessment.

Depending on the frailty-assessment method used, the prevalence of frailty ranged from 55.5% (FIFA) and 60.6% (EFS-C) to 62.5% (gait speed category). Therefore, frailty was defined as either “moderate frailty” or “severe frailty” in the FIFA score classification, as “mild frailty,” “moderate frailty,” and “severe frailty” in the EFS-C or as unfeasible 6MWT or gait speed less than 0.83 m/s. The FIFA score demonstrated the highest predictability of hospital mortality (AUC 0.844, CI 0.656-1.000; *P=*.048) when compared to 6MWT gait speed classification (AUC 0.671, CI 0.487-0.855; *P=*.42) and EFS-C (AUC 0.636, CI 0.254-1.000; *P=*.44) (Table 4, Figure 1).

## Discussion

### Principal Results

This study demonstrates the strong predictive performance of a modern health-monitoring device–based frailty assessment, and, to the best of our knowledge, this is the first trial to investigate the potential of a wearable activity-monitoring device in this specific clinical setting. The main findings of our study were as follows. The FIFA score correctly identifies frail patients, as demonstrated by the strong correlation with baseline serum albumin levels—a well-established biomarker for frailty. Device-recorded preprocedural stress and heart rates in patients with TAVR are independent predictors with increased postprocedural hospital mortality. The FIFA score outperformed conventional frailty assessment methods by correctly identifying patients at higher risk for procedure-related mortality.

The FIFA trial has developed a modern, intuitive, and efficient frailty assessment tool that adds substantially to the growing body of evidence on the relevance of frailty in the selection of patients with TAVR. Its potential in clinical practice is substantiated by its relationship with established measures of frailty such as serum albumin levels, which play a prominent role in the estimation of frailty by reflecting the extent of muscle weakness and malnutrition. Preoperative serum albumin levels <3.5 g/dl have been proven to be an independent predictor of mortality and the inverse and proportional correlation between baseline serum albumin levels and the FIFA score is thus indicative of the assessment tool’s ability to correctly identify frail patients [9].

Furthermore, it has also been shown for the first time that several preprocedural stress parameters (the overall preprocedural stress level, net time in high stress/at rest) are independent predictors of postprocedural mortality. Establishing the link between stress measurements and postprocedural outcomes was enabled by wearable devices and represents a novel finding. The physiological basis for this relationship may be underpinned by the pivotal role that heart rate variability has in deriving the stress parameters. Several studies have verified the association between deviations in heart rate variability scores and postoperative outcomes. The standard deviation of normal to normal intervals has been established as an independent predictor of increased postprocedural mortality in various clinical populations [17-19]. Varadhan et al [20] demonstrated that impaired cardiac autonomic function reflected by a lower heart rate variability is a predictor of frailty and mortality in older women. It is assumed that the underlying pathophysiological process may be the degradation of autonomic control mechanisms, which leads to an overactivated sympathetic system and subsequent neuroendocrine responses in resting stages, thereby hampering physiological recovery. Accordingly, the FIFA trial has also demonstrated that an elevated preprocedural overall maximum heart rate correlates positively with increased hospital mortality. We, therefore, postulate that stress parameters represent a directly measurable surrogate marker of impaired hemodynamic regulation in those patients. Consequently, stress parameters as measured with activity-monitoring devices may help detect high-risk patients and reduce therapeutic futility. Importantly, this information could be combined with other established parameters (eg, albumin levels) in improving the specificity of preoperative risk assessments.

Conventional frailty scores (the EFS-C and the 6MWT gait speed classification) showed limited association with hospital mortality compared to the FIFA score, but given the fact that these criteria were designed to predict long-term outcomes, correlations may improve, as the study progresses over time. In contrast to our findings, gait speed as a single measure was shown to be independently associated with mortality after TAVR procedures. Alfredsson et al [21] reported that the slowest walkers in their study had a 35% higher 30-day mortality than normal walkers, and each 0.2-m/s decrease in gait speed corresponded to an 11% mortality increase. Although the EFS applied in this study has not been subject to validation in a population with TAVR, it demonstrated good predictability of 30-day mortality in older patients after cardiac surgery [22]. The differences in the findings may be attributed to the smaller population examined in this study; however, the ability of the FIFA score to predict short-term mortality within our initial study population is promising. With an AUC of 0.844, it demonstrates excellent discriminatory power [23]. Moreover, not only does it correctly identify frail patients and potentially exceed conventional frailty assessment methods in their predictive ability of postprocedural mortality, but its ease of use and economical approach saving both time and valuable human resources make it an especially attractive tool to use in clinical practice. A further benefit is that while patients may perform differently in a clinical test setting, the gathering of real-life data allows more objective interpretation of patients’ frailty with high interobserver reliability.

### Limitations

There are several limitations to this study. As this is a proof-of-concept study, benchmark data had to be established for comparison with conventional frailty assessment methods; hence, the open-label, nonrandomized, prospective trial design. Owing to the lack of existing data, the concept was established and tested in a relatively small number of patients. Even though the primary endpoint is sufficiently powered, the concept needs to be validated in a larger patient population. Furthermore, the short postprocedural observation period may lead to underestimation of the predictive value of the FIFA score as most frailty-related studies assess long-term outcomes, including 1-year mortality.

### Conclusion

For the first time, the strong predictive performance of wearable health-monitoring device–related assessment compared to that of conventional frailty methods has been shown. The ease of use, objectivity, and high predictive performance may not only save valuable clinical resources but ultimately improve patient selection and safety. The promising initial results warrant further evaluation of the FIFA score classification as a predictor of short-term and long-term mortality after structural heart interventions or conventional surgery.

## Abbreviations

6MWT: 6-minute walk test
AUC: area under the curve
EFS-C: Edmonton Frail Scale classification
EuroSCORE: European system for cardiac operative risk evaluation
FIFA: FItness-tracker assisted Frailty-Assessment
ROC: receiver operating characteristic
STS: Society of Thoracic Surgeons
TAVR: transcatheter aortic valve replacement
VARC: Valve Academic Research Consortium
